# A Literary Curiosity

**Published:** 1882-05

**Authors:** 


### A LITERARY CURIOSITY.

The following rather curious piece of composition was recently placed upon the blackboard at a teachers' institute, and a prize of a Webster's Dictionary offered to any person who would read and pronounce every word correctly. The book was not carried off, however, as twelve was the lowest number of mistakes made in pronunciation:

"A sacrilegious son of Belial, who suffered from bronchitis, having exhausted his finances, in order to make good the deficit, resolved to ally himself to a comely, lenient and docile young lady of the Malay or Caucasian race. He accordingly purchased a calliope and coral necklace of a chameleon hue, and securing a suite of rooms at a principal hotel, engaged the head waiter as his coadjutor. He then dispatched a letter of the most unexceptional calligraphy extant, inviting the young lady to a matinée. She revolted at the idea; refused to consider herself sacrificable to his desires, and sent a polite note of refusal, on receiving which he procured a carbine and a bowie knife, and said he would not forge fetters hymeneal with the queen; went to an isolated spot, severed his jugular vein, and discharged the contents of his carbine into his abdomen. The débris was removed by the coroner."

The mistakes in pronunciation were made on the following words: Sacrilegious, Belial, bronchitis, exhausted, finances, deficit, comely, lenient, docile, Malay, calliope, chameleon, suite, coadjutor, calligraphy, matinée, sacrificable, carbine, hymeneal, isolated, jugular and débris.—*Printers' Circular.*

THE development of the coal resources and industry of Germany has recently received much attention, due, in good part, to the encouraging character of the Hamburg exhibition of that mineral held some time since. It is alleged that the Westphalian coal is superior in heating power to English, and the investigations carried on by some of the most eminent geologists make it appear, though the statement is somewhat surprising, that Germany is in possession of coal fields incomparably more extensive than any that England can show. The Westphalian coal basin alone, it is said, is capable of producing, for seven centuries to come, the same quantity of best coal annually that all England now yields; and not only this, but the basin is not yet fully explored, and is believed to be capable of material extension.

Soda for Burns.—All kinds of burns, including scalds and sunburns, are almost immediately relieved by the application of a solution of soda to the burnt surface. It must be remembered that dry soda will not do unless it is surrounded with a cloth moist enough to dissolve it. The method of sprinkling it on and covering it with a wet cloth is often the very best. But it is sufficient to wash the wound repeatedly with a strong solution. It would be well to keep a bottle of it always on hand, made so strong that more or less settles on the bottom. This is what is called a saturated solution, and really, such a solution as this is formed when the dry soda is sprinkled on and covered with a moistened cloth. It is thought by some that the pain of a burn is caused by the hardening of the albumen of the flesh, which presses on the nerves, and that the soda dissolves the albumen and relieves the pressure. Others think that the burn generates an acrid acid, which the soda neutralizes.

The compulsory provision of life-preservers on steamers, and their manifest utility, suggests the propriety of a law compelling factory owners to provide at each window a cheap and efficient fire-escape, in addition to the appliances and stairways now required. One that would always be ready, easily understood and usable by any person of ordinary intelligence, even under excitement, could be made in the following manner:

To a staple firmly driven in the wall immediately over each window attach a rope, or cord, say three-eighths of on inch in size and long enough to reach nearly or quite to the ground. This cord should be well made and pliable, and might be knotted at intervals of about fifteen inches. The cord should then be rolled into a coil or ball, and tied in place by a small cord or strap ready at a moment's notice to be untied and the end to be thrown out of the window. Men, and even women, could descend it with little difficulty, or the stronger and cool-headed could tie the rope about the bodies of the weaker and quickly lower them to the helpers below.

